# Fmoc-FF hydrogels and nanogels for improved and selective delivery of dexamethasone in leukemic cells and diagnostic applications

**DOI:** 10.1038/s41598-024-60145-z

**Published:** 2024-04-30

**Authors:** Enrico Gallo, Carlo Diaferia, Giovanni Smaldone, Elisabetta Rosa, Giovanni Pecoraro, Giancarlo Morelli, Antonella Accardo

**Affiliations:** 1 IRCCS SYNLAB SDN, Via Gianturco 113, 80143 Naples, Italy; 2 Department of Pharmacy and Interuniversity Research Centre on Bioactive Peptides (CIRPeB) “Carlo Pedone”, University of Naples “Federico II”, Via D. Montesano 49, 80131 Naples, Italy

**Keywords:** Fmoc-FF, Dexamethasone, Peptide-based hydrogels, Nanogels, Leukemia, Diagnostic protocols, Biological techniques, Chemistry, Nanoscience and technology

## Abstract

Dexamethasone (DEX) is a synthetic analogue of cortisol commonly used for the treatment of different pathological conditions, comprising cancer, ocular disorders, and COVID-19 infection. Its clinical use is hampered by the low solubility and severe side effects due to its systemic administration. The capability of peptide-based nanosystems, like hydrogels (HGs) and nanogels (NGs), to serve as vehicles for the passive targeting of active pharmaceutical ingredients and the selective internalization into leukemic cells has here been demonstrated. Peptide based HGs loaded with DEX were formulated via the “solvent-switch” method, using Fmoc-FF homopeptide as building block. Due to the tight interaction of the drug with the peptidic matrix, a significant stiffening of the gel (G′ = 67.9 kPa) was observed. The corresponding injectable NGs, obtained from the sub-micronization of the HG, in the presence of two stabilizing agents (SPAN®60 and TWEEN®60, 48/52 w/w), were found to be stable up to 90 days, with a mean diameter of 105 nm. NGs do not exhibit hemolytic effects on human serum, moreover they are selectively internalized by RS4;11 leukemic cells over healthy PBMCs, paving the way for the generation of new diagnostic strategies targeting onco-hematological diseases.

## Introduction

In the last three decades, supramolecular nanostructures like liposomes, polymeric micelles and fibres, with a size ranging between 10 and 500 nm, have been proposed as potential vehicles for the delivery of anticancer^1–3^ or anti-inflammatory drugs^4,5^, diagnostic agents^6–8^ and nucleic acids^9,10^. The advantages of these supramolecular formulations are related to their ability to alter the in vivo pharmacodynamic and pharmacokinetic properties of active pharmaceutical ingredients (APIs). The delivery of drugs via supramolecular nanoplatforms allows: (i) a significant reduction in drug toxicity, that remains tightly encapsulated within the nanostructures; (ii) a rapid drug clearance via the reticuloendothelial system (RES) and extravasation^11^; (iii) a different biodistribution of the drug, resulting in an increase of the pharmaceutical efficiency^12,13^; (iv) the passive accumulation of anticancer drugs within solid tumors as a consequence of the increased permeability and retention (EPR) effect occurring in the vascular endothelium of cancer and inflamed areas^14^; and (v) the stabilization of therapeutic compounds from degradation and/or inactivation in the bloodstream or macrophages opsonization^15^.

Another benefit provided by nanocarriers is represented by their ability to improve the bioavailability of very low water-soluble drugs, like naproxen, danazol and paclitaxel, thus avoiding the use of not biocompatible organic solvents^16–18^.

Doxil®, a formulation containing DOX hydrochloride, is the first FDA-approved nano drug delivery system based on PEGylated liposome technology. It can be considered as one of the most representative and successful examples of a supramolecular drug formulation, being able to increase API localisation in the tumour site and decrease the most frequent side effects such as myelosuppression and cardiotoxicity, leading to a higher efficacy in tumour treatment compared to the free doxorubicin drug.

Hydrogels (HGs) and nanogels (NGs) are emerging as versatile supramolecular platforms for the delivery of APIs, with growing interest on the part of scientists and pharmaceutical companies.

HGs are three-dimensional space spanning networks made up of hydrophilic building blocks, which may be cross-linked by either chemical irreversible or physical reversible bonds^19^. Deriving from macroscopic HGs progenitors through top-down approaches allowing the obtainment of nano-sized particles to be injected^20^, NGs are composed by a hydrogel-like core structure, enclosed in a surfactant stabilizing shell.

Due to their self-assembling capability, the high biocompatibility, biodegradability and the chemical accessibility, peptides have been proposed as suitable constituents of HGs and NGs^21^. Moreover, peptides offer the feasibility of ad hoc designing their primary sequence and, therefore, the shape, the pore size and the loading/release kinetics of the material^22,23^. The aromatic homopeptide Fmoc-Phe-Phe-OH (Fmoc-FF, *N*^*α*^-9-fluorenylmethoxycarbonyl-diphenylalanine) represents one of the most studied sequences able to self-assemble into macroscopic HGs^24,25^. Starting from Fmoc-FF, we recently developed formulation methods for the obtainment of stable nanogels, in which the fibrillary network of the hydrogel is stabilized in its nanometric form by a couple of surfactants [SPAN®60 (sorbitan monostearate) and TWEEN®60 (polyethylene glycol sorbitan monostearate) in 48/52 w/w ratio]^26^. These formulations were evaluated, in comparison with the corresponding HGs, as systems for the delivery of the hydrophilic, low molecular weight, anticancer drug doxorubicin. DOX loaded HGs and NGs exhibited both a different internalization mechanism and an intracellular distribution in comparison to the free drug^27^. Moreover, we also observed a selectivity of Fmoc-FF NGs towards cancer cell lines overexpressing the protein caveolin-1, such as MDA-MB-231 cells^28^.

Here, we further investigate the versatility of these formulations as potential delivery systems for the vehiculation of the drug dexamethasone (DEX) that, due to its very low solubility in water (89 µg/mL), has been subclassed as a drug of class III by the Biopharmaceutical Classification System (BCS)^29^. DEX is a synthetic cortisol widely used to treat a variety of diseases, including ocular disorders, cancer, autoimmune diseases, and recently for COVID-19 infection^30^. The low solubility of DEX and the severe side effects (e.g. hyperglycemia, ulcers and hydro-electrolytic disorders), related to the systemic administration, hampered its clinical use. For all these limitations, the development of an efficient DEX-loaded nanoformulation may be currently of great interest for several biomedical applications. DEX filled HGs and NGs were studied in terms of encapsulation efficiency, shelf stability, drug release and in vitro activity on B-cells leukemia cell model RS4;11. Moreover, the ability of Fmoc-FF nanogels to penetrate leukemic cells has been evaluated, as well as the hemotoxicity and the stability in human serum using FITC filled nanoformulation. Finally, we also tested the propensity of these NGs to be selectively internalized by leukemic cells.

## Materials and methods

### Materials

Lyophilized Fmoc-FF powder was purchased from Bachem (Bubendorf, Switzerland). Dexamethasone (DEX) was purchased from Sigma Aldrich (Milan, Italy). TWEEN®60, SPAN®60, and all other chemicals and solvents were obtained from commercial sources and used as received unless otherwise stated. All the solutions were prepared by weight using doubly distilled water.

### Formulation of Fmoc-FF empty HGs and NGs

Fmoc-FF HGs and NGs were formulated according to previously described procedures^27^. Fmoc-FF hydrogels were obtained at a concentration of 1.0 wt% using the “solvent-switch” method. Briefly, a Fmoc-FF stock solution (100 mg/mL) in dimethyl sulfoxide (DMSO) was prepared. To obtain the hydrogel, 60 µL of the solution were diluted with 540 μL of double distilled water under vortexing (7 s). The metastable suspension, undergoing an opaque-to limpid transition, was left aging for 5 min. The macroscopic formation of a self-supporting hydrogel was assessed via the inverted test tube assay. Nanogels were obtained using a top-down sub-micronization strategy. Fmoc-FF hydrogel, prepared as previously reported, was homogenized at 35,000 min^−1^ for 5 min into 3.4 mL of an aqueous solution containing TWEEN®60/SPAN®60 at a *w/w* ratio of 52/48 (total of 3.0·10^–5^ mol), using a MICCRA GmbH D9 homogenizer. The obtained suspension was then tip sonicated at 9 W for 5 min using a Branson SFX250 homogenizer.

### Formulation of Fmoc-FF DEX filled HGs and NGs

Drug loading was permitted thanks to the modification of the previously described formulation procedures. In details, DEX powder was dispersed in DMSO at a concentration of 15.3 mmol/L (200 mg/mL) and 30 µL of this solution were added to 30 µL of a DMSO Fmoc-FF solution (200 mg/mL). The sample was vortexed to obtain a homogeneous mixture and then diluted with 540 μL of double distilled water under stirring (7 s). The suspension was left to gel for a few minutes at room temperature. To obtain the DEX filled NG, the HG thus prepared was subjected to the top-down methodology as previously described. Purification of NGs formulation from the non-encapsulated/free quote of drug was achieved via size exclusion chromatography (SEC) on a pre-packed column Sephadex G-50 pre-equilibrated with water. The drug loading content (DLC), delineated as g_DEX encapsulated_/g_(surfactant+peptide)_, and the encapsulation ratio (ER%), defined as the weight percent of DEX amount in the NG on the total drug embedded during formulation, were assessed, after calculating g_DEX encapsulated_ as the difference between the total amount of DEX added during the procedure and the free DEX fraction. The drug concentration was analytically calculated using a UV–Vis Thermo Fisher Scientific Inc (Wilmington, Delaware USA) Nanodrop 2000c spectrophotometer equipped with a 1.0 cm quartz cuvette (Hellma) using a molar absorptivity (ε) of 14,900 mol^−1^L cm^−1^ at λ = 239 nm.

### Formulation of FITC filled NG

FITC-filled NG was obtained using the method described above. In details, 30 µL of Fmoc-FF solution (200 mg/mL in DMSO) and 30 µL of FITC-CNS solution (12.8 mmol/L in DMSO) were diluted with 540 µL of water and the obtained HG was subjected to the top-down procedure. The amount of FITC encapsulated (50 μmol/L) was calculated by subtracting the amount of the free dye from the total amount of the loaded one. The FITC content was determined by UV–vis spectroscopy through calibration curves acquired by measuring the absorbance at λ = 492 nm (ε = 75,000 mol^−1^L cm^−1^).

### Thioflavin T (ThT) spectroscopic assay

The ThT assay was carried out on xerogels of DEX filled HG. A coverslip glass was sprinkled with a layer of macroscopic HG and left to dry overnight. The dried sample was stained for 30 s with 50 µL of a 50 µmol/L ThT water solution. The excess of the dye was removed with a filter paper and then the sample was dried overnight. Images of the stained xerogel were acquired through a Leica DMRB microscope equipped with a fluorescence Leica DFC320 video-camera (Leica, Milan, Italy) with a 20 × objective and a Green Fluorescent Protein (GPF) filter. The analysis was carried out using the software Image J (National Institutes of Health, Bethesda, MD).

### Circular Dichroism (CD) characterization

Far-UV CD spectra of DEX filled and empty HGs and NGs were collected on a Jasco J-810 spectropolarimeter supplied with a NesLab RTE111 thermal controller unit at 25 °C. In details, 80 µL of hydrogel or nanogel formulations were placed in a 0.2 mm quartz cell. The measurements were recorded at a wavelength range between 320 and 190 nm. Other experimental settings were the following: scan speed = 20 nm·min^−1^, time constant = 4 s, sensitivity = 10 mdeg, bandwidth = 1 nm. Each spectrum was obtained by averaging three scans and reported in optical density (O.D.). Origin 2018 (OriginLab Corporation, version 8.1) software was used to arrange the comparative CD analysis.

### Fourier transform infrared (FT-IR) spectroscopy

FT-IR spectra of filled and empty HGs and NGs were captured using a Jasco FT/IR 4100 spectrometer (Easton, MD) in an attenuated total reflection (ATR) mode and using a Ge single-crystal at a resolution of 4 cm^−1^. Data were refined using a built-in software. Spectra were obtained performing a total of 300 scans with a rate of 2 mm·s^−1^ against a KBr background.

### Dynamic light scattering (DLS) measurements

The hydrodynamic radii (R_*H*_) and the zeta potential (*ζ*) of NGs were obtained through Dynamic Light Scattering (DLS) using a Zetasizer Nano ZS (Malvern Instruments, Westborough, MA, USA). Instrumental settings for the measurements were a backscatter detector at 173° in automatic modality, room temperature, and disposable sizing cuvette as cell. The DLS acquisitions were carried out in triplicate on aqueous samples after centrifugation at room temperature at 13,000 rpm, for 5 min. Data of ζ were acquired as the average of 20 measurements.

### Nanoparticles tracking analysis (NTA) measurements

The NTA measurements were done using a Nanosight NS300 (Alfatest, Italy). NG formulations of Fmoc-FF filled with DEX and FITC were diluted 1000-fold in double-distilled water to a final volume of 1 mL. The dilution was performed according with the ideal particle per frame value (20–100 particles/frame). The parameters were selected based on the manufacturer’s software manual (NanoSight NS300 User Manual, MAN0541-01-EN-00, 2017).

### Rheological characterization

Rheological measurements of empty and DEX filled Fmoc-FF HGs were performed at 25 °C using a rotationally controlled stress rheometer (Malvern Kinexus) equipped with a 15.0 mm flat-plate geometry (PU20:PL61). For each experiment, a freshly made hydrogel sample (360 μL) at a concentration of 1.0 wt% was exploited and placed in a humidity chamber. A gap of 1.0 mm was employed throughout the measurements. To identify the regime of linear viscoelasticity preliminary strain (0.01–100%) and oscillatory frequency (0.01–100 Hz) sweeps were carried out. Time-sweep oscillatory tests (in a 0.1% strain and 1.0 Hz frequency regime) were run for 20 min. Final analyses are presented as G′ (storage elastic modulus)/G″ (shear loss or viscous modulus) ratio in Pascal [Pa].

### Drug release from HGs and NGs

DEX release from HGs was assessed by using a conic tube (1.5 mL) with 400 µL of drug filled hydrogels (1.0 wt%) and adding 800 µL of 0.100 mol/L phosphate buffer above them. At each time-point, 400 µL of this solution were collected and replaced with an equal amount of fresh phosphate buffer. UV–vis spectroscopy was used to calculate the extent of free DEX. All the release experiments were conducted in triplicates. The amount of the released DEX was expressed as a percentage of the ratio between the amount of the released drug and the total quantity of drug initially loaded into HGs. The DEX release from NGs was achieved, after being prepared and purified from free DEX, by adding 9.0 mL of phosphate buffer to 1.0 mL of DEX-NG suspension. From this solution, left under stirring at 37 °C, at each time point up to 72 h, 1.0 mL was removed and restored with an equal fresh amount of PBS. Then 400 µL of this solution were purified from free DEX via size exclusion chromatography (SEC) on a pre-packed column Sephadex G-50 pre-equilibrated with water. The amount of the released DEX was quantified, after lyophilization, by deducting the free DEX from the total amount of loaded DEX. The DEX concentration was calculated by UV–vis spectroscopy using calibration curves created by detecting absorbance at λ = 239 nm. All the release experiments were run in triplicates. Similar to HGs, DEX release profile from NGs was provided as percentage of released drug/total drug loaded into NGs.

### Cell culture and blood components separations

The human lymphoblastic leukemia cell line RS4;11 was obtained from the IRCCS SYNLAB SDN Biobank^31^. Cells were detained in IMDM medium, supplemented with 10% FBS, 10 mM Glutamine and the addition of 1 × Penicillin–Streptomycin solution (Lonza) and kept in a humidified incubator at 37 °C and 5% CO_2_. For each experimental procedure, RS4;11 cells were used within 10 passages from thawing, in exponential growth phase. To ensure the absence of mycoplasma contamination, cells were regularly tested using the Mycoblue Mycoplasma Detector kit (Vazyme).

Freshly withdrawn venous blood, anti-coagulated with Ethylenediaminetetraacetic Acid (EDTA) (1.8 g/L), was collected from healthy volunteers and immediately processed (within 1 h). After dilution with PBS 1×, red blood cells (RBCs) and serum were separated by centrifugation (2000×*g*, 6 min). After five washing with PBS 1×, RBCs were diluted 1/20 to obtain 5% hematocrit. Peripheral blood mononuclear cells (PBMC) were collected by Ficoll-Paque gradient centrifugation (Pancoll® density 1077 g/L, PanBiotech, Aidenbach, Germany) at 400×*g* for 30 min.

### Hemotoxicity assay

RBCs suspension was added with increasing amount of Fmoc-FF nanoparticles (*n* = 1.3·10^10^, 2.6·10^10^ and 1.3·10^11^ particles/mL; where *n* was estimated by NTA measurements). The mixtures were then vortexed for 30 s and incubated for 8, 24 and 72 h in static conditions at room temperature. At the end of the incubation time, each sample was gently vortexed and centrifuged (9000×*g*, 3 min). PBS and water were utilized as positive and negative controls, respectively. The acquired supernatants were assessed by spectrophotometric investigation at λ = 577 nm through Victor Nivo (Perkin Elmer, Waltham, Massachusetts, USA).

### NGs stability in serum

The stability of NGs was evaluated using the Nanosight NS300 (Alfatest, Italy). 100 µL of FITC filled NG suspension were incubated with 900 µL of human serum and the mixture was placed under stirring at 37 °C up to 72 h. At different time points, 100 µL of the solution were collected and 1000-fold diluted in double-distilled water to a final volume of 1 mL. The solution was then analyzed in order to obtain the number of fluorescently labeled nanoparticles *per* milliliter and to assess their dimensions. The settings were chosen as above reported.

### Cells viability

RS4;11 cell viability was assessed using physical parameters using the cytofluorimeter (Cytoflex V2-B4-R2 (Beckman-Coulter, CA, USA). Leukemic cells ability to internalize Fmoc-FF FITC filled NGs was assessed over time by flow cytometry. In details, FITC filled NG suspension (*n* = 1.3·10^10^ particles/mL) was added to 1.6·10^4^ RS4;11 cell medium and incubated for different increasing times (0, 15, 30, 60 and 120 min). At each time-point, cells were collected, centrifuged (3000 rpm × 5 min at RT) and the supernatant, containing non internalized nanoparticles, was discarded. Subsequently, cells were washed twice with PBS and resuspended in a final volume of 200 µL of PBS for further processing.

### NGs internalization

The propensity of FITC filled NGs to be specifically internalized in leukemic cells was evaluated by mixing the same number of PBMC to RS4;11 (1 × 10^5^ cells) pre labelled with the following antibodies mix: CD45-KO, CD19-PC7, CD3-PC5, CD56-APC700 and HLA-DR-PB. Flow cytometry experiments were conducted analysing on the FITC channel a minimum of 10,000 recorded events using the Cytoflex V2-B4-R2 (Beckman-Coulter, CA, USA) instrument. Three independent replicates were used for each time point. To ensure reduced nanoparticles toxicity, the percentage of viable cells over the total was also observed by flow cytometry using morphological parameters. As a qualitative representation of the internalization process, an aliquot of treated cells from the same samples previously analysed were plated on a µ-Slide 8 Well high ibiTreat (Ibidi) and observed by confocal microscopy, using a 63 × water-immersion objective on the MICA microhub (Leica Microsystems) platform. For each ROI, the same conditions of exposure time and contrast were used. The corresponding contrast phase images were also acquired as reference. Hoechst (H3570, Thermo) and HLA-DR-PC5 antibody (A07793, Beckman coulter) were used for the nuclei and membrane stain of RS4;11, respectively.

## Results and discussion

### Formulation of DEX filled HGs and NGs

**Figure 1:**
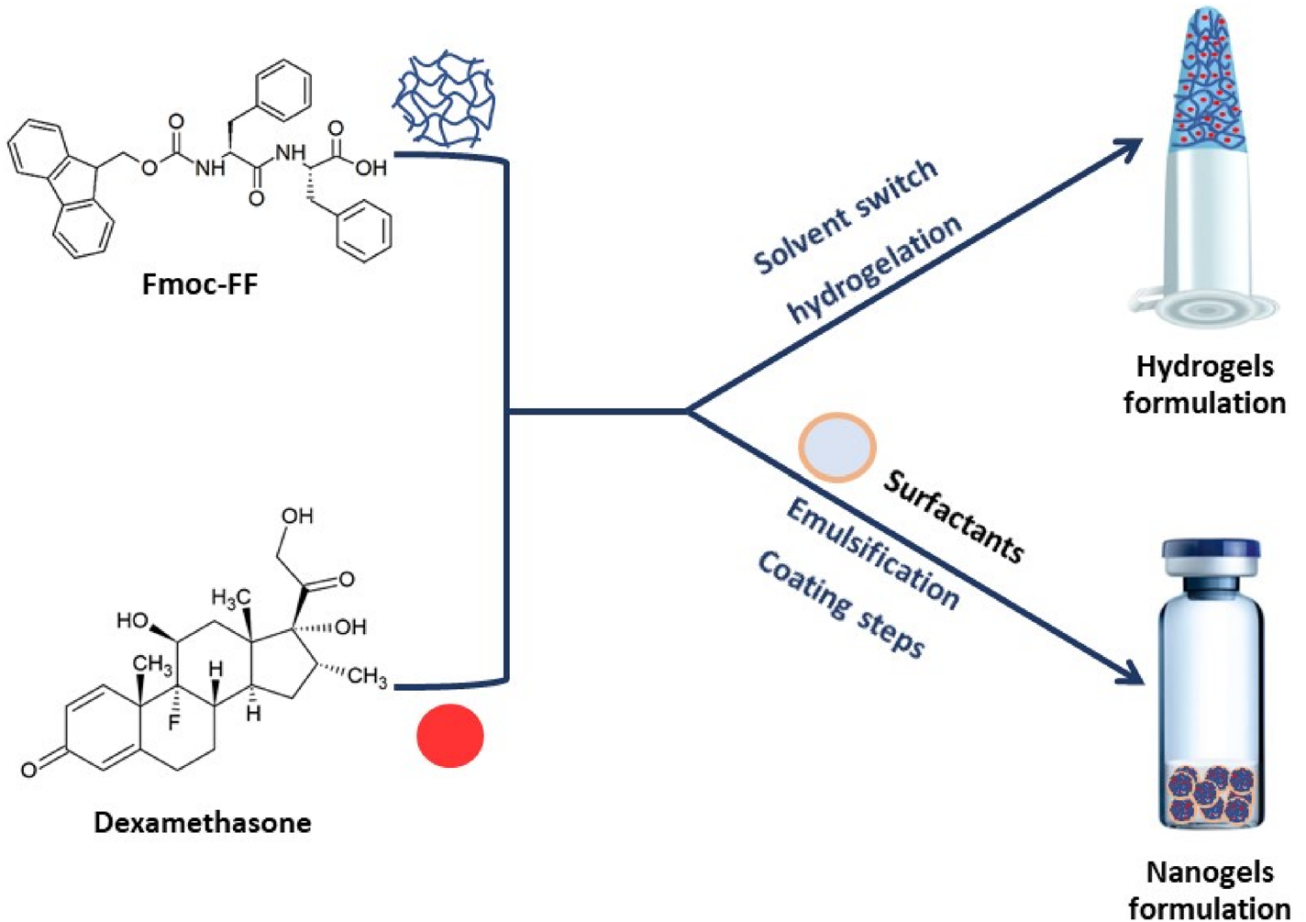
Schematic representation of components and methodologies for the formulation of peptide HGs and NGs. Chemical formula of Fmoc-FF and DEX are also reported.

**Figure 2:**
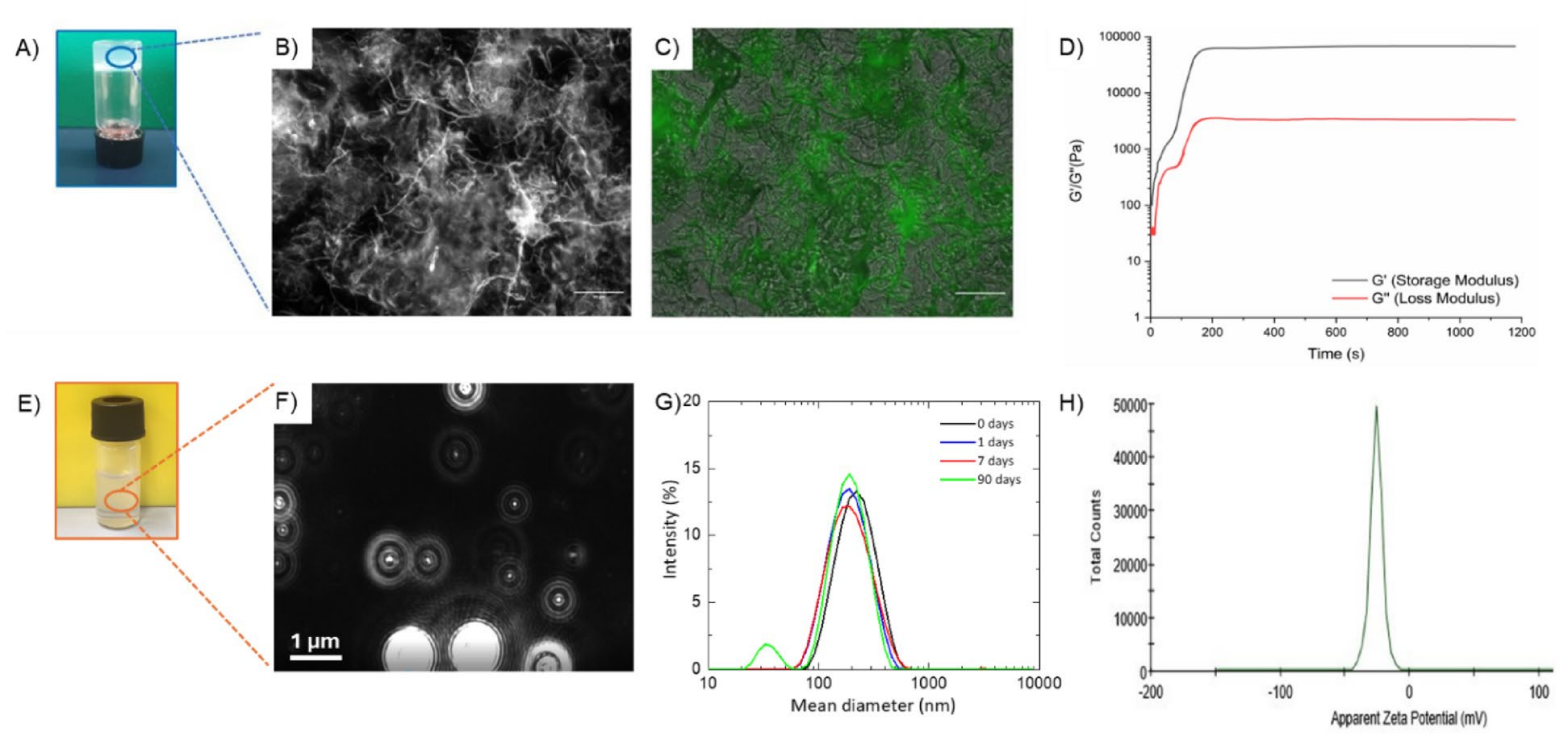
Structural characterization of Fmoc-FF DEX filled HG and NG. (**A**) Inverted test tube for DEX filled HG. (**B**) Optical microscope images of Thioflavin T (ThT) stained HG in the brightfield and (**C**) in the green (GFP, Green fluorescent protein; λ_exc_ = 488 nm, λ_em_ = 507 nm) spectral region. The scale bar is 50 µm. (**D**) Rheological profiles of time sweep measurements. G′ and G″ are graphed in grey and red, respectively. (**E**) Vial containing the DEX loaded nanogel suspension and (**F**) a representative video frame of the DEX filled NG recorded by NTA. Intensity profile (**G**) and Z potential (**H**) of DEX filled NG measured by DLS technique. DLS profiles are reported over time up 90 days: freshly prepared (blue line), 7 days (red line) and 90 days (green line).

Fmoc-FF is able to generate a macroscopic HG by the physical entanglement of fibers via a multiscale organization process, triggered by a switch of the starting conditions (pH or solvent)^24,25^. Structural and molecular dynamic simulation studies by Uljin and co-workers revealed that the single fibers in the gel have a nanocylindrical architecture due to the interlocking of four twisted anti-parallel β-sheets through lateral π-π interactions^32^. This organization allows the coexistence in the final matrix of both a hydrophobic and a hydrophilic compartment, which may potentially accommodate APIs having different solubilities. The corresponding injectable NG formulation was then prepared by submicronization (homogenization and tip sonication) according to the top-down method^21^. This procedure requires the addition of surfactants (TWEEN®60 and SPAN®60) to improve the stability of the colloidal suspension. From the experimental point of view, it was firstly evaluated the capability of the Fmoc-FF hydrogel to entrap DEX (Fig. 1). The loading of the drug was achieved by the solvent-switch method, according to the same procedure used for the preparation of the empty HGs. Stock peptide solution in DMSO (200 mg/mL) was mixed in an equal volume (30 μL) with the DEX solution (200 mg/mL in DMSO). Gelation was then triggered by dilution in water (gel at 1.0 wt%, 10 mg/mL final concentration), followed by vortexing for 7 s. Likewise the empty Fmoc-FF formulation, also DEX-filled matrix is completely formed after an opaque-limpid transition without significant modifications of the gelation kinetics (2 min). The inverted test tube (Fig. 2A) confirms the self-supporting behaviour of the filled HGs. Moreover, no syneresis can be detected, thus allowing to assume that all the initial DEX (6.0 mg) is entrapped in the matrix. DEX loaded HG was then converted by submicronization (homogenization and tip sonication) in its injectable formulation, the DEX filled NG. During this procedure the starting HG preparation is diluted with a solution containing a mix of surfactants that allows to stabilize the formulation. At the end of the top-down procedure, we obtained a slightly opaque suspension that scatters the light. The amount of DEX encapsulated into the peptide-NG was found, after purification by gel-filtration, to be 1.82 mg/mL with an encapsulation ratio ER% of 90.8%. This amount of DEX encapsulated in nanoparticles allows to obtain a drug concentration ~ 20-fold higher than the free drug solubility in water.

### Structural characterization of DEX Filled HGs and NGs

Hydrogels and nanogels filled with DEX were fully characterized from the structural point of view by using a set of techniques like optical microscopy, rheology, Dynamic Light Scattering (DLS), Nanotracking Analysis (NTA), Circular Dichroism (CD) and Fourier Transform Infrared (FTIR). Representative optical images acquired in the bright field for DEX loaded xerogels (see Fig. 2B) show that the sample is dominated by networks of unbranched fibrillary elements mutually interconnected. This structuration is typically reported for Fmoc-FF and other peptide-based hydrogels^33,34^. The specific organization of Fmoc-FF makes it responsive to different colorimetric dyes, too. For this reason, a Thioflavin T (ThT) fluorescence assay on xerogels was performed. ThT (also known as Basic Yellow 1 or Methylene yellow) is a benzothiazole salt widely employed to visualize and quantify both in vitro and in vivo the presence of misfolded protein aggregates, including amyloid or plaques^35^. ThT is formed by a benzothiazole and a benzylamine rings connected through a carbon–carbon bond, able to free rotate in solution. This rotation produces a quenching of all the excited states generated by excitation. On the contrary, when it binds rich in β-sheet structures, the two rotational rings planes are mutually blocked, thus producing fluorescence signal. A strong green emission (Fig. 2C) can be detected for Fmoc-FF xerogel containing DEX (GFP filter) stained with a solution of ThT, suggesting the presence of β-secondary structure elements. To mechanically characterize DEX filled HG, a rotational rheological analysis was performed, allowing to study the viscoelastic features of DEX containing matrix. Preliminary evaluation of the optimal measurement parameters was performed via dynamic oscillation strain sweep (at a frequency of 1.0 Hz, Fig. S1A) and dynamic frequency sweep (at ω = 0.1% strain, Fig. S1B), reporting all the data as G′ (storage modulus) and G″ (loss modulus). The linear viscoelastic region (LVR) was identified in the 0.01 < ω < 4.8%. The time sweeps oscillatory measurements (20 min, 1.0 Hz and 0.1% strain, Fig. 2D) were carried out on DEX filled HG, prepared as above described. As clearly visible from the G′ (67.9 kPa) > G″ (3.33 kPa) value, our sample is analytically in the gel state. The tanδ (G′/G″ = 20.4) also suggests a prominent viscoelastic nature of the gel with a creation of a very strong matrix. It was already reported that the combination of a different building block into Fmoc-FF can alter its mechanical response. It was also demonstrated that multicomponent HGs of Fmoc-FF with hexapeptides exhibit an increased mechanical response^21^. In this specific case, the inclusion of DEX in Fmoc-FF matrix strongly increases the Gˈ value. This result can be probably attributed to the tight interactions of the hydrophobic drug with the peptide.

DEX filled NG suspension was structurally characterized by DLS technique. DLS intensity profiles and the zeta potential distribution revealed the presence of nanoparticles with a mean diameter, zeta potential (ζ) and polydispersity index (PDI) of 203.5 ± 90.0 nm, − 25.6 ± 1.2 mV and 0.170, respectively (Fig. 2G,H). The size of nanoparticles and, as a consequence, also their charge, does not change significantly over time after 90 days. NG suspension was also characterized using NTA analysis, which allows to both determinate the size of the single particle distributions and quantify the number of particles in solution. From the inspection of the size distribution, reported in Fig. S2, it can be observed that most of the nanoparticles have a diameter of ~ 105 nm, and only a few parts of them have a bigger size. However, the 90% of the size distribution is lower than 187.1 ± 12.3 nm, being in agreement with the Gaussian curve found from the DLS profile (Fig. 2G).

**Figure 3:**
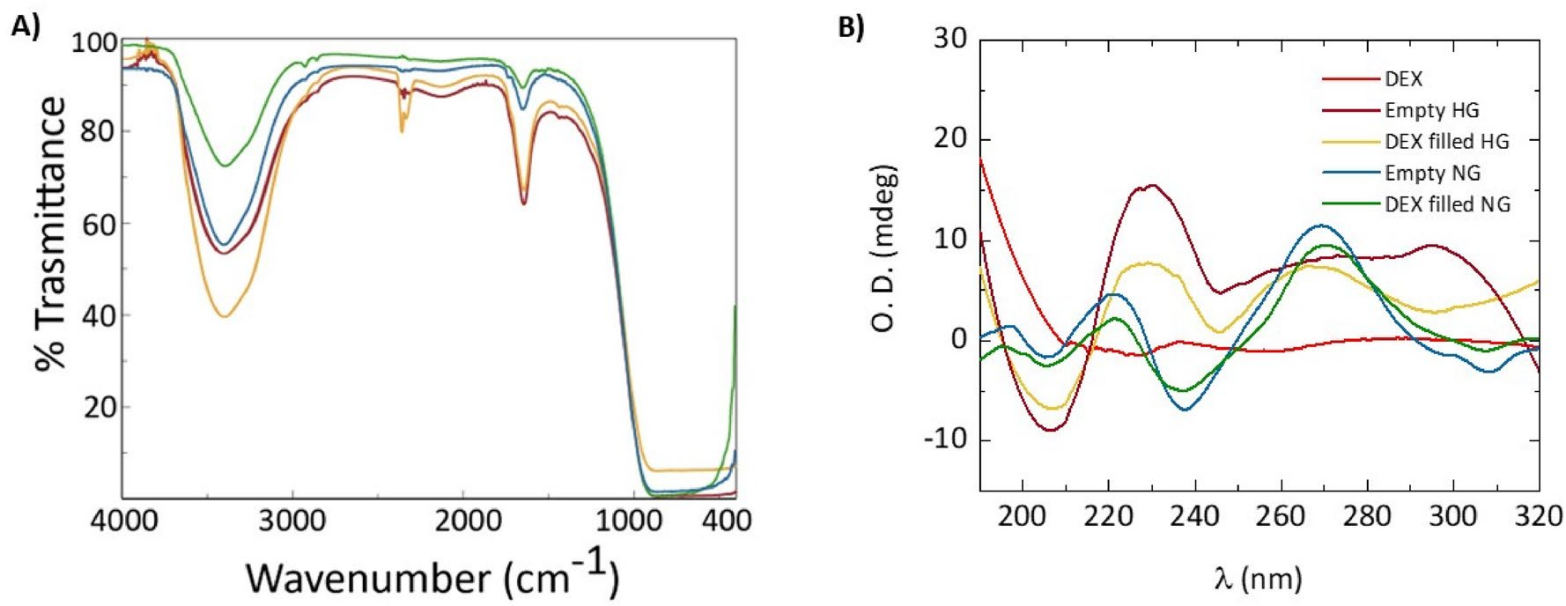
Secondary structure analysis of Fmoc-FF HGs and NGs. (**A**) Comparison between FT-IR spectra in 4000–400 cm^−1^ range of empty and DEX filled HGs and NGs. (**B**) Comparison between CD spectra of empty and DEX filled HGs and NGs recorded between 320 and 190 nm. The mutual analysis of JASCO raw data (jws files) was obtained using Origin 2018 software.

Secondary structuration analysis of both HGs and NGs was performed recurring to FTIR and CD spectroscopies. In Fig. 3, FTIR spectra of empty and DEX filled HGs and NGs are collected. Independently from the considered samples, all the systems share a common transmittance signature. No differences, excluding the intensity, can be detected between empty and filled HGs and NGs, highlighting the common Fmoc-FF structuration as supramolecular element for all the systems. Additionally, FTIR makes evident that the DEX encapsulation (at the explored concentrations) does not alter the general organization of the peptide, both in HGs and NGs. For peptide-based aggregates in water, FTIR spectra are typically governed by two main signals, a broad one in amide A region (3700–3000 cm^−1^) centred at ⁓ 3400 cm^−1^ and a second sharper band in amide I region (1700–1600 cm^−1^) around 1641 cm^−1^. The amide A signal is attributed to the water exposure of aggregates, generated by both symmetric and asymmetric stretching of O−H and N−H functional groups. A deconvolution of this region, carried out to evaluate the contributions of N−H involved in H− bond interactions with respect to free N−H, did not allow to discriminate between the two contributions. The carbonyl stretching motus, responsible for the band at 1641 cm^−1^, also suggests the presence of β-sheets rich assemblies.

These considerations are reinforced by a comparative CD analysis performed in the 190–320 nm acquisition range and reported in Fig. 3B as optical density (O.D.). In Fig. S3 the singular recorded spectra are reported. Far UV-CD spectra of both empty and filled formulations are characterized by common peaks, with a bathochromic effect for hydrogels systems. The negative signal (238 and 243 nm for HGs and NGs, respectively) can be generally referred to the presence of β-sheet structuration of the peptide building block in the supramolecular system. The broad signal (less evident for empty HGs) centred, for all the spectra, at 268 nm may be interpreted as the typical signature of the Fmoc moiety^26^. Again, DEX evidently does not alter the Fmoc-FF architecture, both in HGs and NGs.

### Drug release from HG and NG

**Figure 4:**
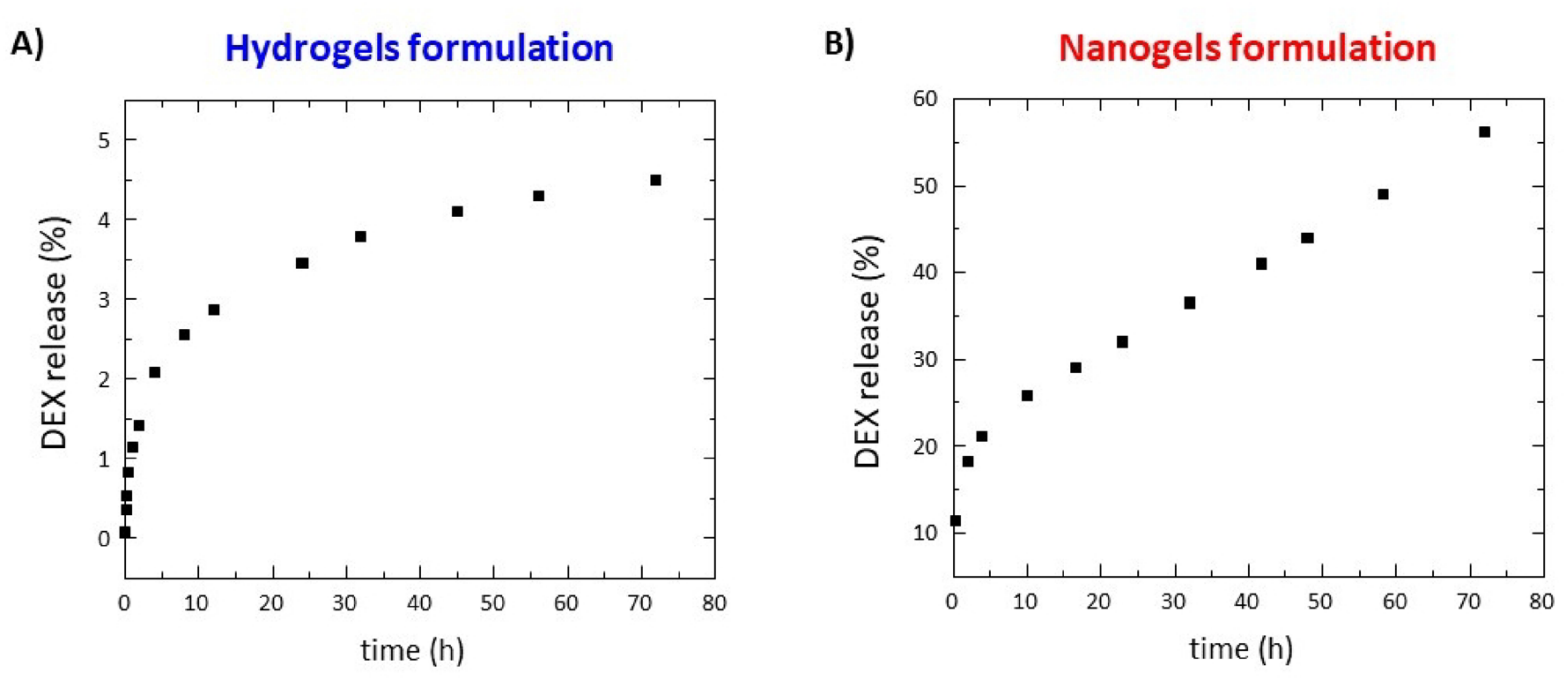
Drug release (%) profiles for: (**A**) Fmoc-FF DEX filled HG; (**B**) Fmoc-FF DEX filled NG.

The in vitro DEX release from HG and NG was evaluated in a 0.100 mol/L phosphate buffer solution up to 72 h. The release profiles for both supramolecular systems are described in Fig. 4A,B. Due to the distinct macroscopic state of HG and NG, two different approaches were used for the quantification of DEX release. For HG release, DEX filled HG was prepared into a conic tube and put in contact with a double volume of the solution. A small amount of PBS was cyclically replaced with an equal volume of the fresh one. Instead, for the second case, NG was entirely (1 mL) placed into PBS solution at 37 °C immediately after their preparation, purification, and quantification of the amount of DEX encapsulated. The DEX released was evaluated through UV–vis spectroscopy by measurement of the absorbance at λ = 239 nm. The extent of drug was reported as a percentage of the ratio between the amount of released drug and the total drug initially loaded. As expected, the drug release kinetics is very different between HG and NG with a percentage of ~ 4% and ~ 60% released, respectively after 72 h. In Fig. 4B it can be observed a sustained and constant release of the drug over time, which leaves to predict a complete release (100%) after 160 h. The extremely slow-release profile of the HG can be attributed to the strong interaction occurring between the peptide and the drug into the matrix, as also suggested by the very high stiffness of DEX filled HG. However, it is important to note that the result of the release could be partially attributable to the different experimental procedures used, according to the literature, for HGs and NGs. Indeed, in the release of drug from the HG the solution is in contact with only one HG surface, whereas for the NG samples, nanoparticles are completely surrounded by the medium. On the other hand, the starting concentration of DEX is higher in the HG respect to the NG (4.0 mg in 400 µL and 1.82 mg in 1.0 mL, respectively). Looking at the literature it can be concluded that kinetics profiles of our HG is similar to that of other peptide hydrogels, which allows a sustained release of the drug only after several days^36,37^.

### NG stability in serum and hemotoxicity

**Figure 5:**
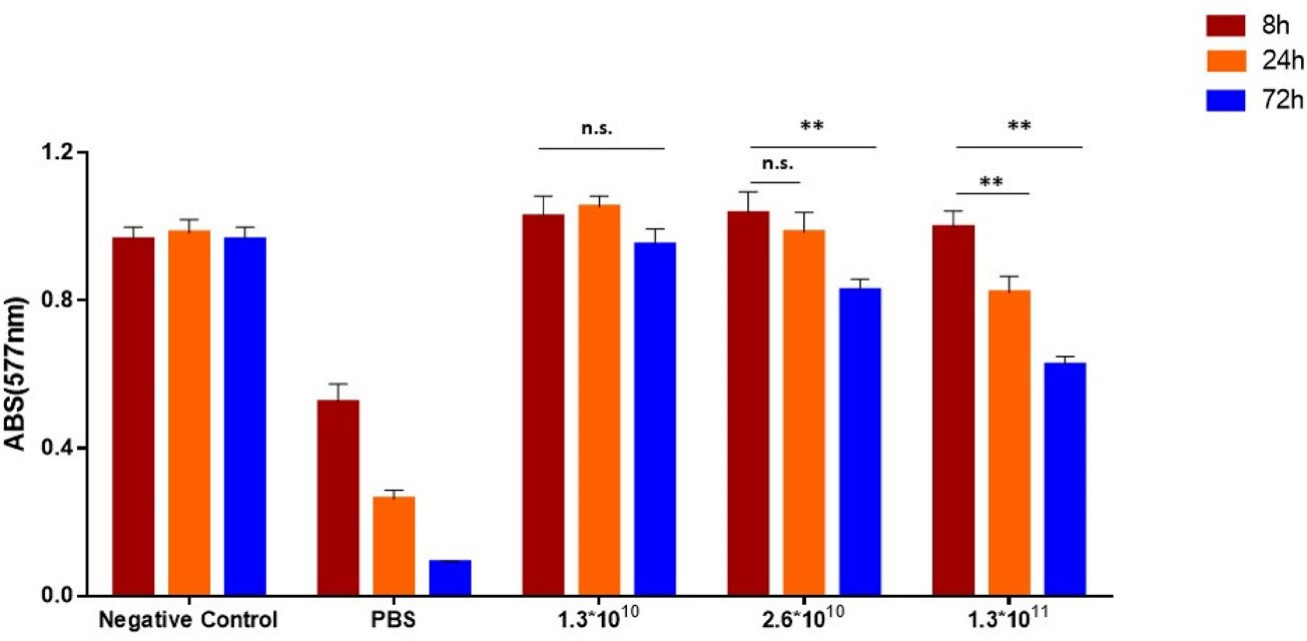
Quantitative hemotoxicity evaluation (Abs at 577 nm) by spectrophotometric analysis of different concentration of Fmoc-FF NG in terms of number of nanoparticles *per* mL of solution, after 8 h (garnet bars), 24 h (orange bars) and 72 h (blue bars) of incubation. **p-value < 0.01. n.s. = not significative, Unpaired T-test. Error represents standard deviation (SD) of three independent experiments.

In order to evaluate the potential in vivo employment of peptide based NGs, we studied their stability upon incubation with human serum (1/10, v/v) at 37 °C. NG stability was followed by NTA analysis over time. The analysis was performed using a NG formulation encapsulating FITC, a fluorescent dye, which allows to selectively distinguish NG particles from proteins and others aggregates commonly present in the serum. As shown in Fig. S4, no significant variations can be detected on the size and on the number of particles up to 72 h. Moreover, hemotoxicity assay was also performed to check NG compatibility with biological fluids. The experiment was simply performed by incubating the empty NG with a RBCs suspension. Three different NGs concentrations, in terms of nanoparticles number (*n* = 1.3·10^11^, 2.6·10^10^ and 1.3·10^10^ particles/mL), were studied. As shown in Fig. 5, Fmoc-FF nanoparticles exhibit hemotoxicity after 24 and 72 h at *n* = 1.3·10^11^ and 2.6·10^10^ particles of NG for mL, respectively. On the other hand, no hemotoxic effect was observed at the lower tested concentration (*n* = 1.3·10^10^ particles/mL) even after 72 h of incubation. Therefore, we decided to use the non-hemotoxic concentration for subsequent experiments. The lack of hemotoxic effect of Fmoc-FF NG makes them optimal for possible use in direct injection into the bloodstream. Indeed, for procedures performed on patients, this capability makes nanogel cuts compatible with all pre-diagnostic contrast medium infusion procedures or even with possible drug administration directly via the vein^38^.

### Cytotoxicity assays

**Figure 6:**
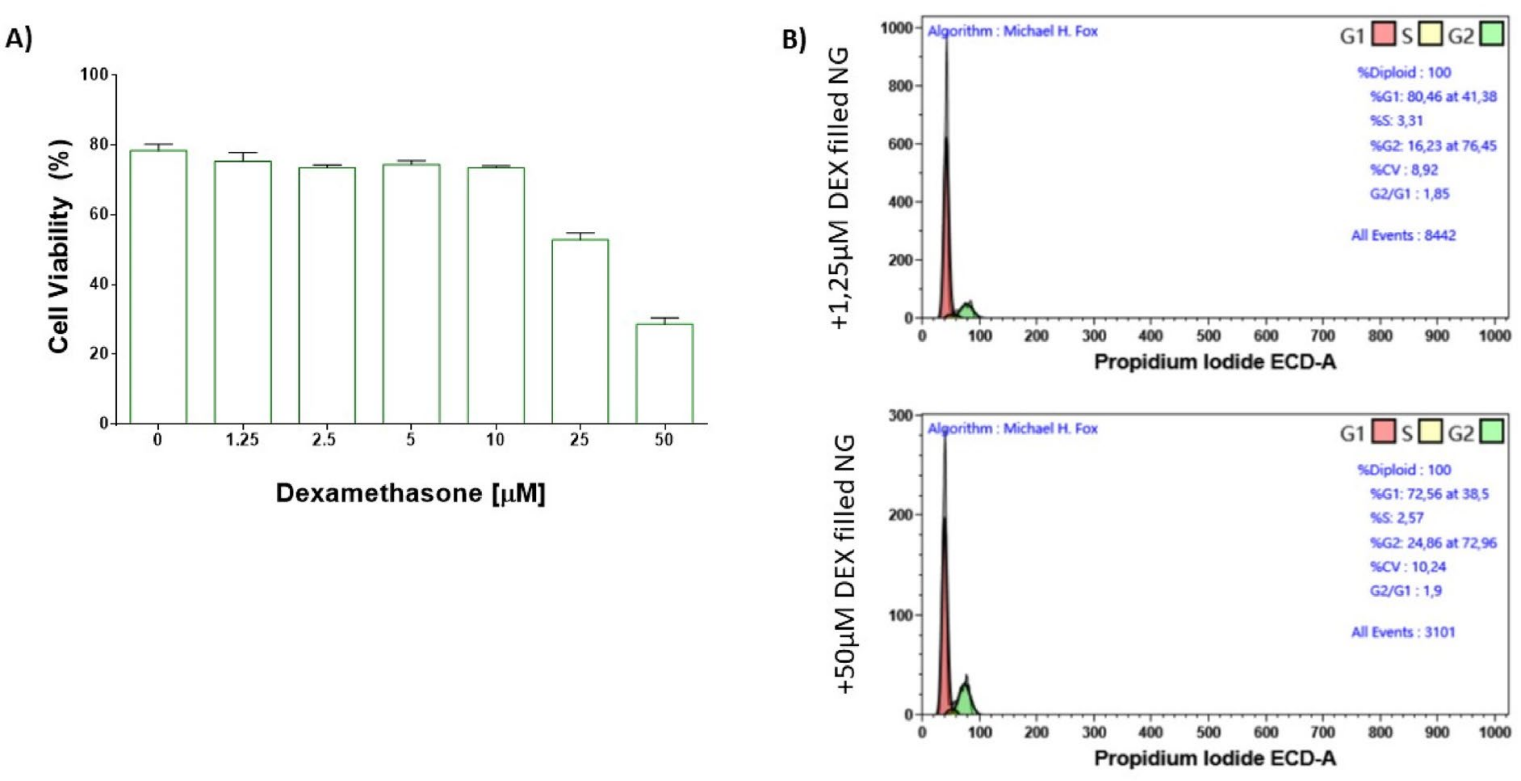
(**A**) Cell viability evaluation of RS4;11 cells treated with Fmoc-FF DEX filled NG at different concentration after 24 h of incubation. (**B**) Flow cytometry analysis of the cell cycle distribution in RS4;11 treated with Fmoc-FF DEX filled NG at 1.25 µM (upper panel) and 50 µM (lower panel) after 24 h of incubation. Cell cycle phases were calculated using Michael H. Fox algorithm.

The in vitro performances of DEX filled NG was evaluated on the B-cells leukaemia cell model RS4;11, known to be particularly sensitive to DEX treatment^39^. Initially, the half maximal inhibitory concentration (IC_50_) of the formulation was assessed by incubating cells for 16 h with NGs filled with increasing drug concentrations. Cell viability was then determined by cytofluorimetric analysis. The gating strategy to distinguish live and dead cells was reported in the supplementary material (Fig. S5). Results in Fig. 6A indicate that the calculated IC_50_ value is ranged between 25 and 50 μM of the encapsulated DEX. Release kinetics studies indicate that, after 24 h, the percentage of released DEX is around 3.5% for HGs, and around 32% for NGs.

This toxicity is due only to the drug; indeed, no cytotoxic effect was detected upon cell incubation with the same number of empty nanoparticles (data not shown). In addition, we analysed the cell cycle of cells treated with DEX filled NG and observed a reduction in the G1 phase with a subsequent increase in the G2-M phase following the treatment (Fig. 6B), which is also associated with the dose of the analysed DEX filled NG (Fig. S6). These experiments show that DEX filled NGs have the ability to correctly release APIs, making them excellent candidates for the development of new nanovectors for diagnostic and/or therapeutic use.

### Uptake of fluorescent NGs

**Figure 7:**
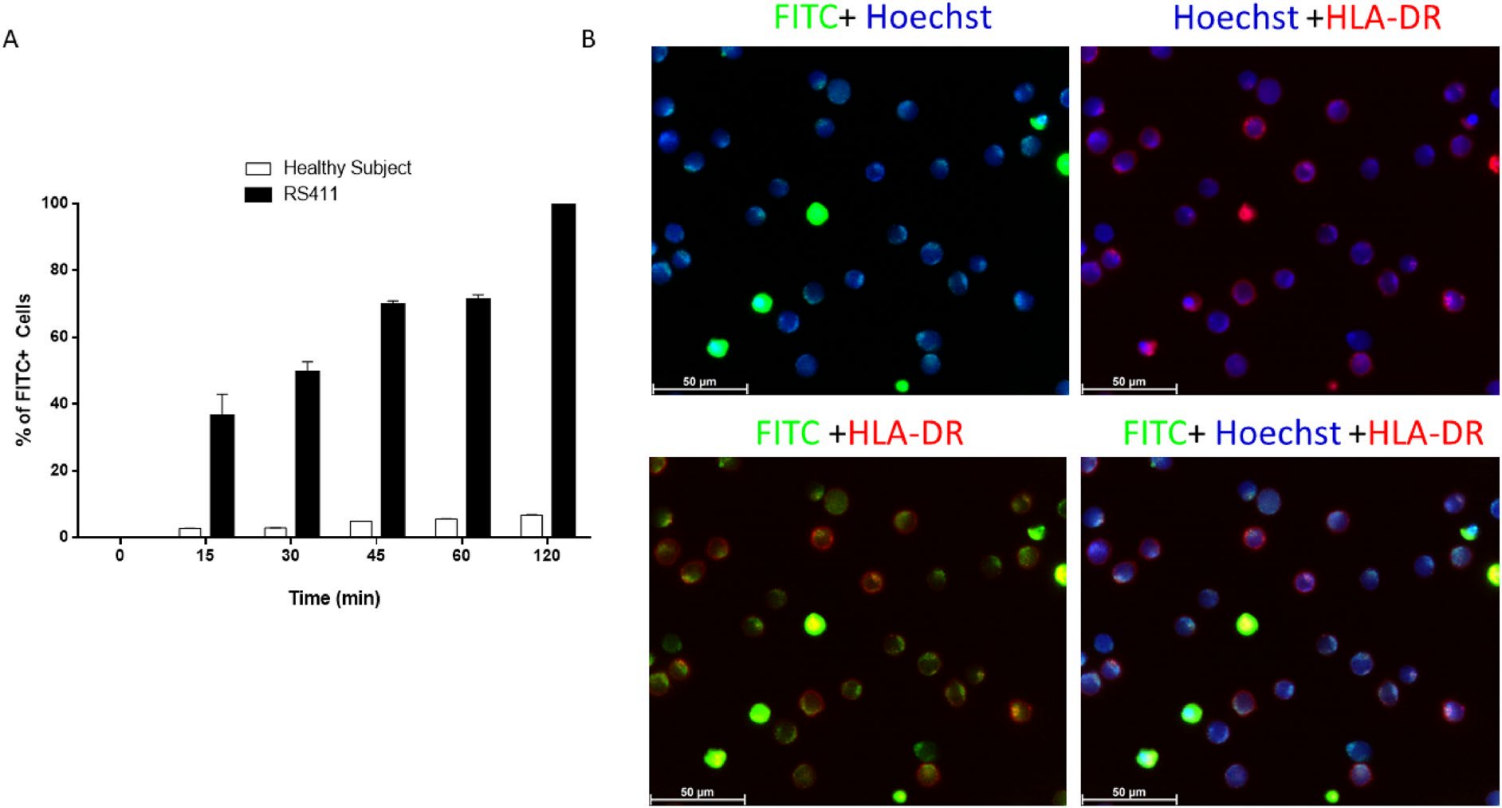
(**A**) Bar chart plot of percentage of positive-FITC cells after incubation with Fmoc-FF FITC filled NG of PBMC derived from healthy subject (white bars) and RS4;11 cells (black bars). Error represents Standard Deviation (SD) of three independent experiments. (**B**) Qualitative representation, by fluorescence microscopy, of Fmoc-FF FITC filled NG internalization in RS4;11 after 120 min of incubation. Magnification 63x. Scale bar 50 µm.

**Figure 8:**
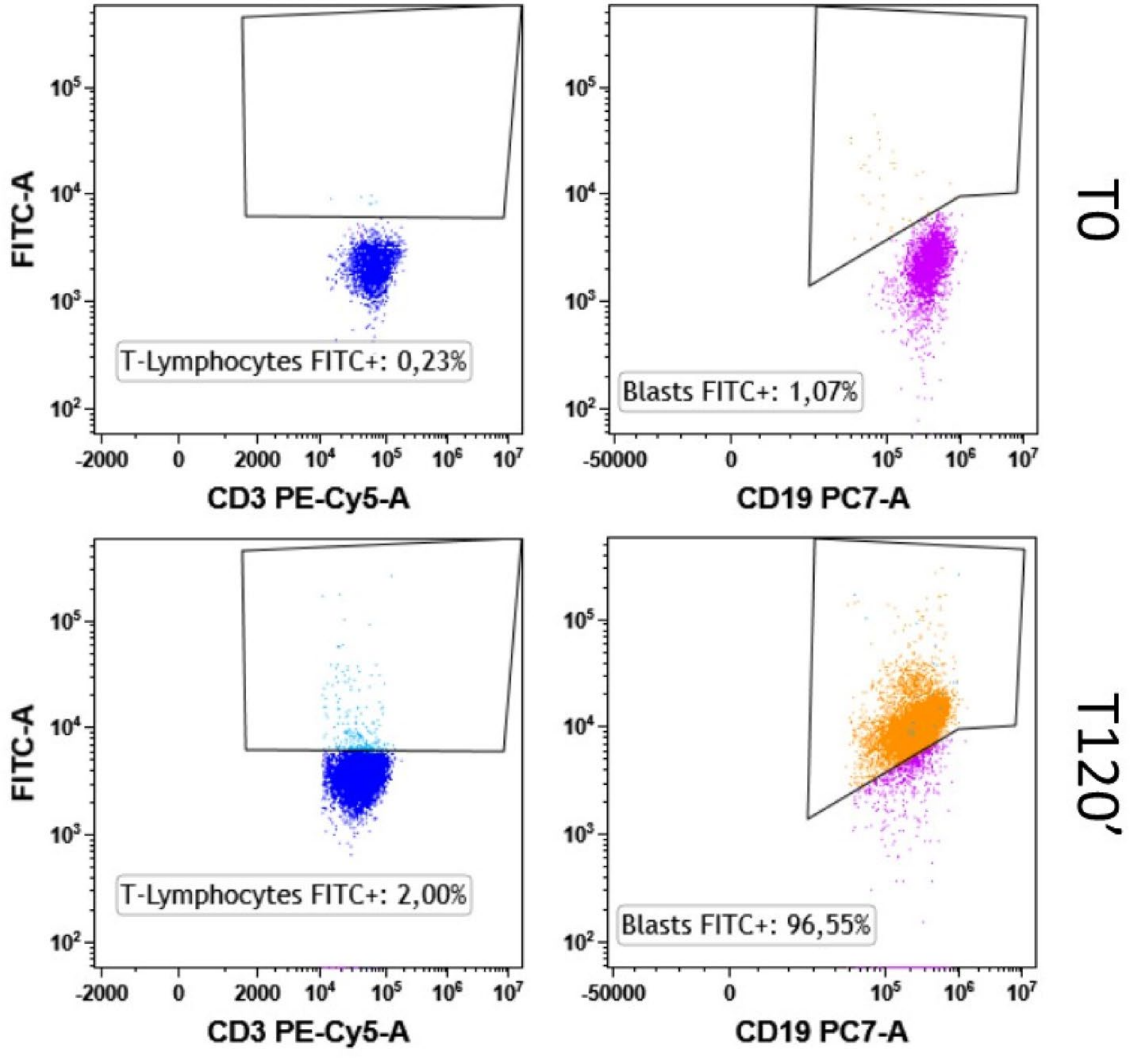
Dot plot cytofluorimetric analysis (FITC vs CD3-PC5 and FITC vs CD19-PC7) of Fmoc-FF FITC filled NG internalization in T-Lymphocytes from healthy subject (left) and RS4;11 cells (right) at T0 (upper panels) and after 120 min of incubation (lower panels). Numbers report the percentages of FITC positive cells.

To assess the ability of NGs to specifically penetrate into leukemic cells, an internalization assay was performed and followed by cytofluorimetric analysis, due to the loading of a fluorescent dye, FITC, into the NG (n = 1.3·10^10^ particles/mL). As shown in Fig. 7, RS4;11 gradually internalized NGs over time, showing 100% positivity to FITC signal after 120 min of incubation (Fig. 7A), whereas in cells of a healthy volunteer patient, nanoparticles do not enter in significant rates (Fig. 7A, white bars). Interestingly, after only one hour of incubation, FITC positivity exceeded 60%, indicating a good internalisation capacity of NGs. Internalisation was also confirmed by confocal microscopy experiments. As it can be seen from Fig. 7B and Fig. S7, after 120 min incubation RS4;11 cells are able to internalise the FITC-filled NGs in the thin cytoplasmic ring, as clearly shown by the merge of the FITC signal with that of Hoechst (nuclear marker) and HLA-DR (membrane receptor present on RS4;11 cells). The rapid internalisation capacity of Fmoc-FF NGs by leukaemic cells makes them extremely effective for the development of new diagnostic and therapeutic strategies targeting onco-haematological diseases. Indeed, it is crucial to be able to identify all leukaemic cells, i.e. the Minimal Residual Disease^40,41^ so as to be able to prevent any relapses that are often lethal for the patients. For this reason, having a system capable of marking circulating leukaemic cells would make it possible to develop diagnostic and subsequently therapeutic strategies aimed at eliminating minimal residual disease. Finally, the ability of NGs to be exclusively internalised by leukaemic cells was assessed in a healthy blood sample to which the same number of leukaemic cells (RS4;11) were added. Leukaemic cells were identified by pre-marking them with CD19-PC7 and HLA-DR-PB450, compared to healthy T lymphocytes marked with CD3-PC5 (Gating strategy in Fig. S8). As it can be seen from Fig. 8, after 2 h of incubation the leukaemic cells (right panels) completely incorporate the FITC-filled NGs, compared to the T-Lymphocytes of healthy volunteer (left panels), which, on the contrary, show negligible percentages of interlayering. There are two strategies for targeting nano-vectors in cancer cells, meaning the exploitation of active or passive internalisation mechanisms^42^. While active targeting has many positive aspects related to the possibility of specifically targeting tumour cells, there are several difficulties in obtaining functionalised nano-vectors, mainly related to specificity of action and costs. Passive targeting, on the other hand, exploits the distinctive features of the tumour environment compared to healthy tissue, so as to favour the internalisation of the nano-vector into the tumour microenvironment^43–45^. Passive targeting of AML by different types of nanoparticles has been widely explored as a solution to vehicle drugs preferentially into diseased cells over healthy ones^46^. For example, a liposomal-encapsulated cytarabine and daunorubicin formulation named CPX-351, approved both by FDA and EMA for the treatment of newly diagnosed therapy-related AML and/or AML with myelodysplasiarelated changes^47^, has shown preferential accumulation in AML cells over normal cells^48^. Moreover, Chandran et al. demonstrated that their PLGA polymer–protein core–shell formulation of nanoparticles, loaded with a mixture of everolimus, sorafenib, and inhibitors of mTOR, MAPK, and STAT5, produced a drastic synergistic lethality against leukemic cells, without affecting the viability of normal blood cells^49^. We previously demonstrated that Fmoc-FF NGs can be passively internalized by breast cancer cells through a caveolin-mediated mechanism^28^. Overexpression of caveolin-1 has been often seen as a distinctive marker for different leukemic subtypes^50,51^. In particular, abnormal expression of caveolin-1 has been observed in adult T-cell leukemia^52,53^. For this reason, we hypothesise that abundant caveolin-1 expression in leukemic cells may be the reason why Fmoc-FF NGs selectively enters diseased cells over the healthy ones. Altogether, this could pave the way for the possibile use of these nanogels for the development of diagnostic protocols aimed at identifying leukaemic cells specifically, perhaps by internalising labelled probes within them^54^. Obviously, future functionalisation studies could allow Fmoc-FF NGs to be used for other oncological pathologies as well, using special reference targets towards which the nanogels could be directed.

## Conclusions

Peptide-based nanoplatforms like HGs and NGs have been recently proposed as alternative vehicles for the delivery of different therapeutic or diagnostic agents. The advantages of building up the nanosystem using peptides, is the possibility to modulate the properties of the vehicle, both in terms of morphology, structural properties and in terms of their ability to interact with the host molecule. Moreover, peptides also offer the possibility to insert two or more functions in the same sequence, thus allowing to obtain a supramolecular system able to interact at the same time with both hydrophobic and hydrophilic drugs. For instance, Fmoc-FF HGs and NGs can encapsulate with high efficiency both hydrophilic drugs like doxorubicin and hydrophobic ones like dexamethasone. Our group has already demonstrated the ability of Fmoc-FF NG to encapsulate doxorubicin^27^ and here we used the same NG to encapsulate DEX. Data show the ability of the nanogel to properly retain and release DEX enough to result in leukemic cell death. Furthermore, it was shown that Fmoc-FF NG has no hemotoxic activity and can internalise into leukemic cells very quickly, which would make them promising systems for in vivo applications. Finally, Fmoc-FF NGs are able to specifically internalise into leukemic cells in a mixed sick cell/healthy cell system.

This result paves the way to the possible use of these platforms for the simultaneous delivery of at least two drugs or reporter molecules, with different features. To date, two strategies have been developed for targeting nano-vectors in cancer cells: either by active or passive internalisation^55^. Although active internalization has the main advantage of precisely targeting tumour cells by exploiting surface epitopes binding, there are several difficulties in obtaining functionalised nano-vectors, like off-targeting issues and relatively high costs. Passive targeting, on the other hand, is facilitated by some of the distinctive features of the tumour environment when compared to healthy tissue, like its propension to better absorb circulating nanoparticles^44,56,57^. Moreover, different types of leukemic cell types overexpress caveolin-1, previously identified as the main entrance gate for Fmoc-FF nanoparticles in cancer cells^28,52,53^ making them sensibly more prone to endocite NPs. Given that, data obtained with Fmoc-FF nanogels, albeit preliminary, suggest a possible use of these nanocarriers to passively target leukemic cells over healthy PBMCs with a good specificity in the bloodstream. This strategy, ultimately, may serve as the most suitable tool for the development of new diagnostic protocols aimed at the detection of blood tumours, even at minimal residual disease level, for example by internalising labelled probes targeting specific genetic mutations within Fmoc-FF nanoparticles^54^. In conclusion, peptide based nanogels could represent a valid alternative to liposomes for drug delivery through a passive targeting mechanism, so the NGs herein proposed seems to represent a very promising system for the selective delivery of DEX in leukemic cells.

### Supplementary Information

Supplementary Figures.

## Data Availability

The datasets used and/or analysed during the current study available from the corresponding author on reasonable request.
