# Supplementary material for: Fmoc-FF hydrogels and nanogels for improved and selective delivery of dexamethasone in leukemic cells and diagnostic applications
Source: Sci Rep. 2024 Apr 30;14:9940. doi: 10.1038/s41598-024-60145-z (PMC11061151; doi:10.1038/s41598-024-60145-z)
Supplement: Supplementary file 1 — Supplementary Figures. [file 41598_2024_60145_MOESM1_ESM.pdf]

Supplementary Materials

Fmoc-FF hydrogels and nanogels for improved and selective delivery of Dexamethasone in leukemic cells

Enrico Gallo,[1, †] Carlo Diaferia, [2,†] Giovanni Smaldone,[1] Elisabetta Rosa,[2] Giovanni Pecoraro,[1] Giancarlo Morelli,[2] Antonella Accardo[2,*]

[1]IRCCS Synlab SDN, Via Gianturco 113, Naples, 80143, Italy

[2]Department of Pharmacy and Interuniversity Research Centre on Bioactive Peptides (CIRPeB) "Carlo Pedone", University of Naples "Federico II", Via D. Montesano 49, 80131 Naples, Italy.

† These authors have contributed equally

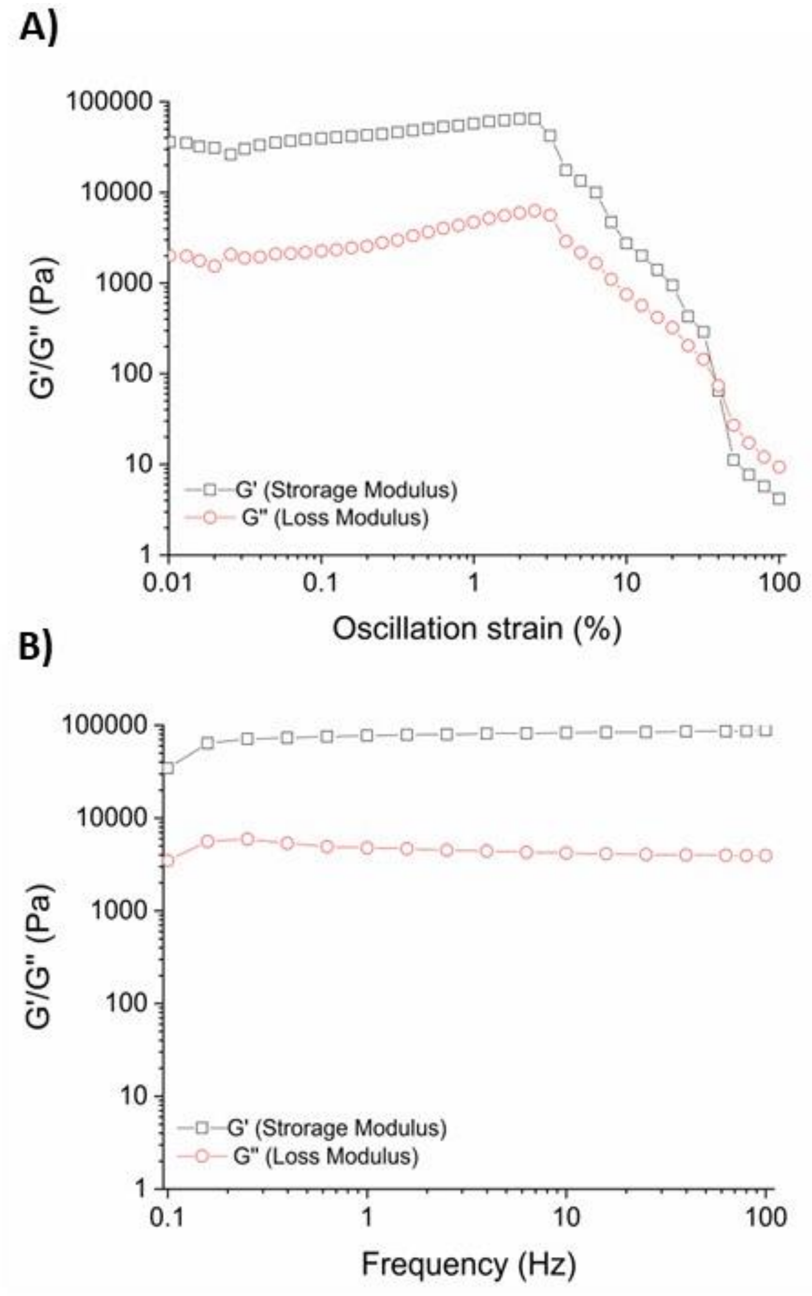

Figure S1. Oscillation strain sweep test(A) and frequency sweep test (B) for DEX filled HG.

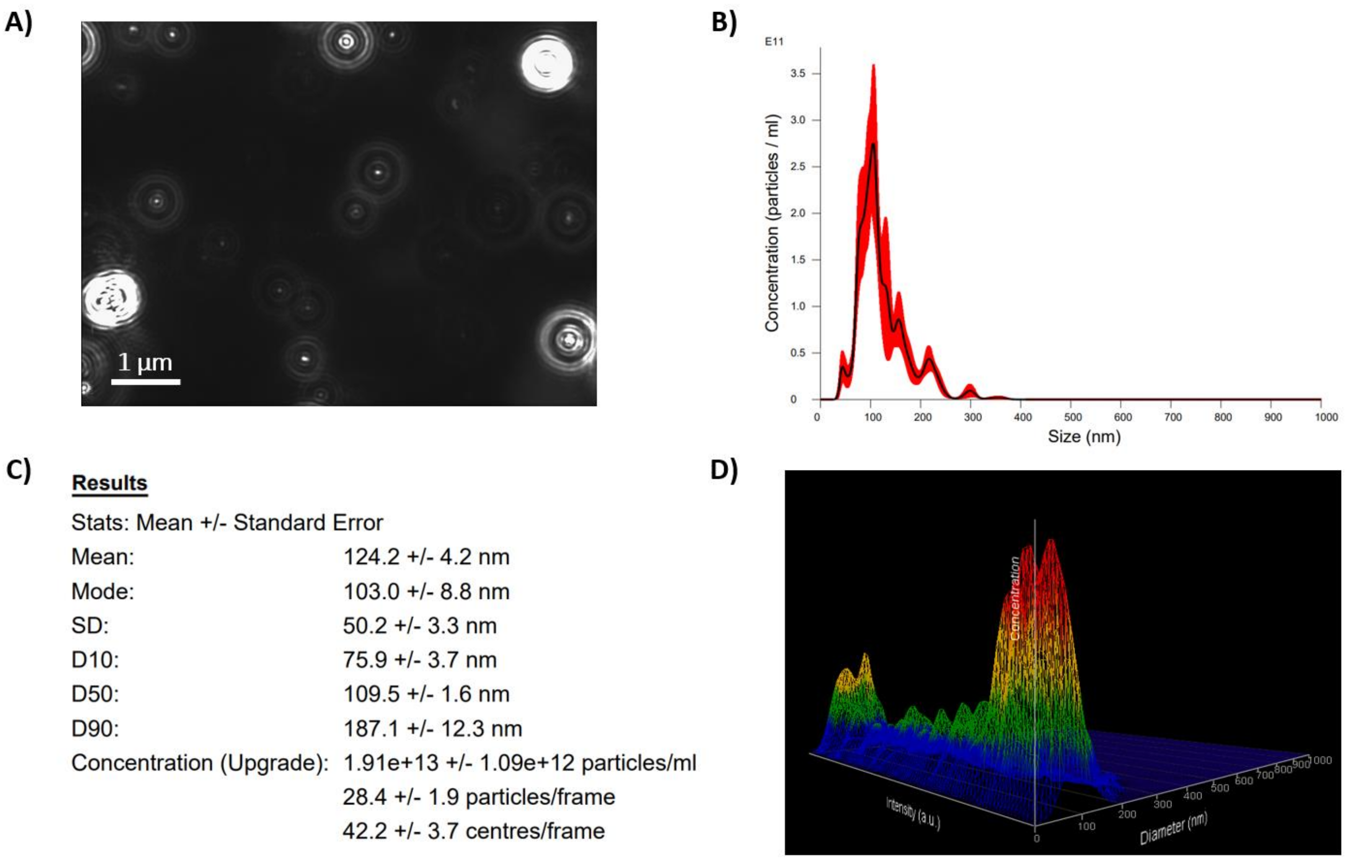

Figure S2: (A) Representative video frame of the Fmoc-FF DEX filled nanogels. (B) Size distribution of particles using nanoparticle tracking analysis (NTA). (C) Results of the analysis. (D) Representative 3D graph (particles concentration vs. intensity vs. diameter).

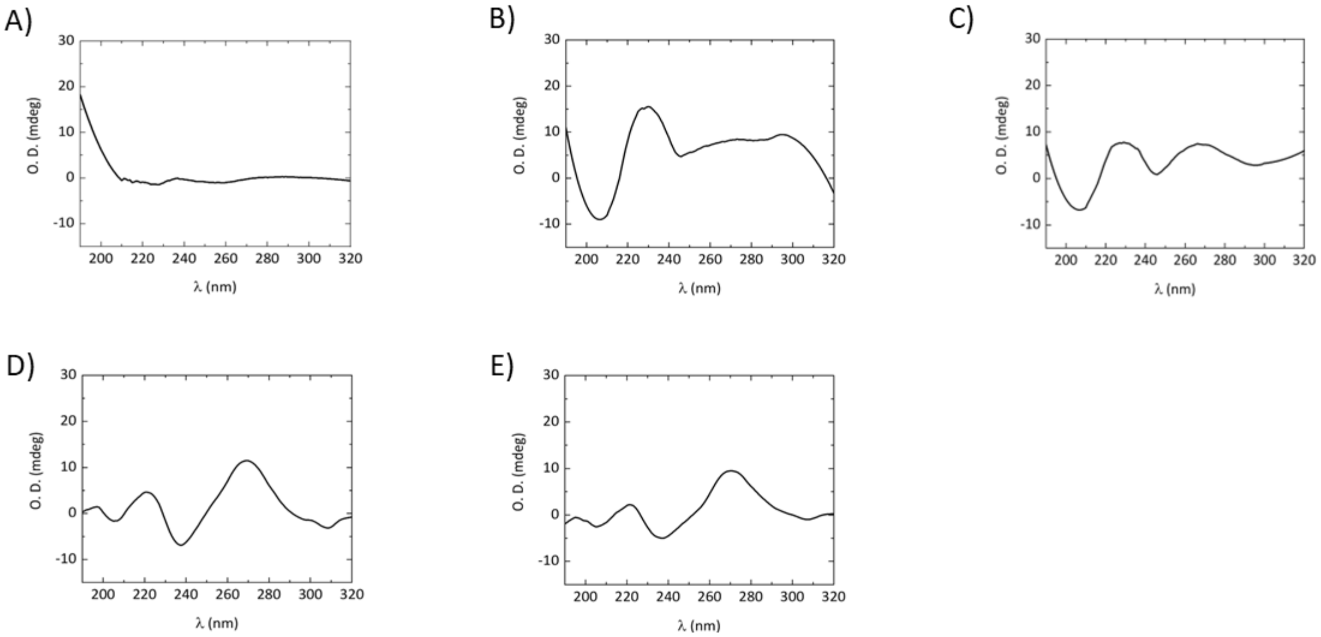

Figure S3. CD spectrum of DEX (A), empty HG (B), DEX filled HG (C), empty NG (D), DEX filled NG (E).

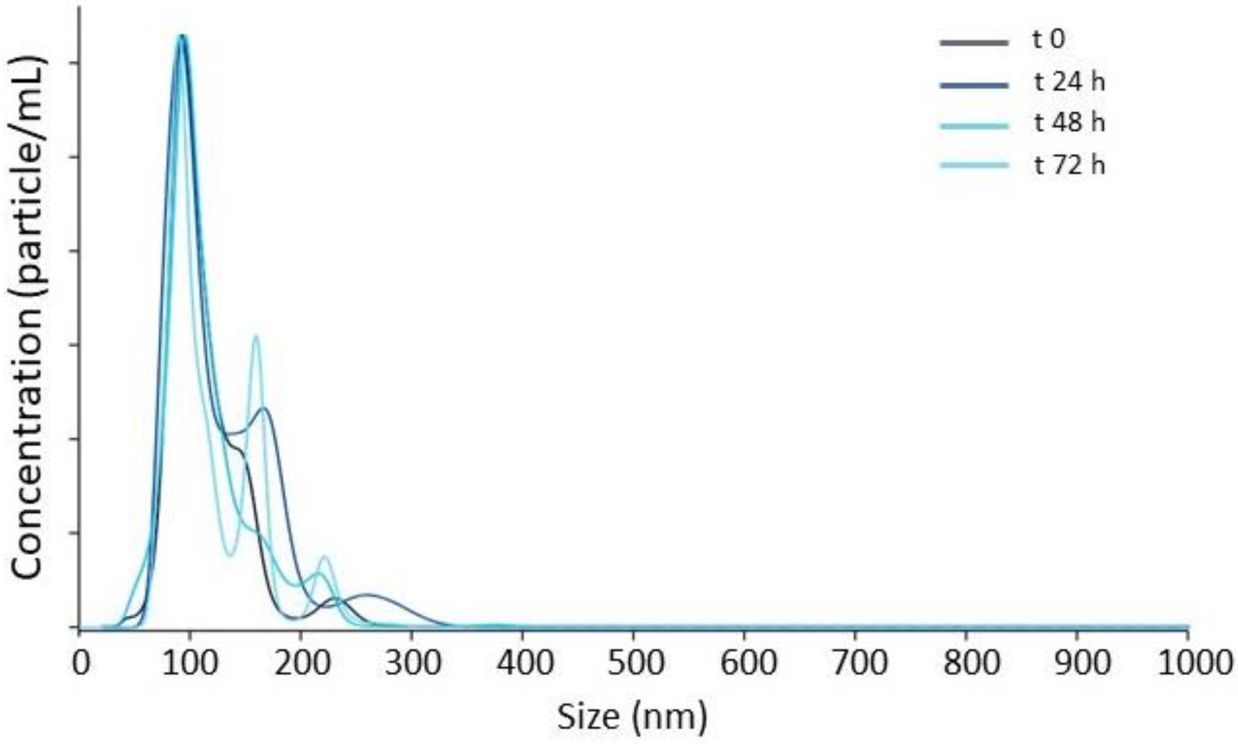

Figure S4: Nanoparticle tracking analysis carried out on FITC filled NG freshly prepared (grey line) and after incubation in human serum at 24 hours (dark blue line), 48 hours (blue line) and 72 hours (light blue line).

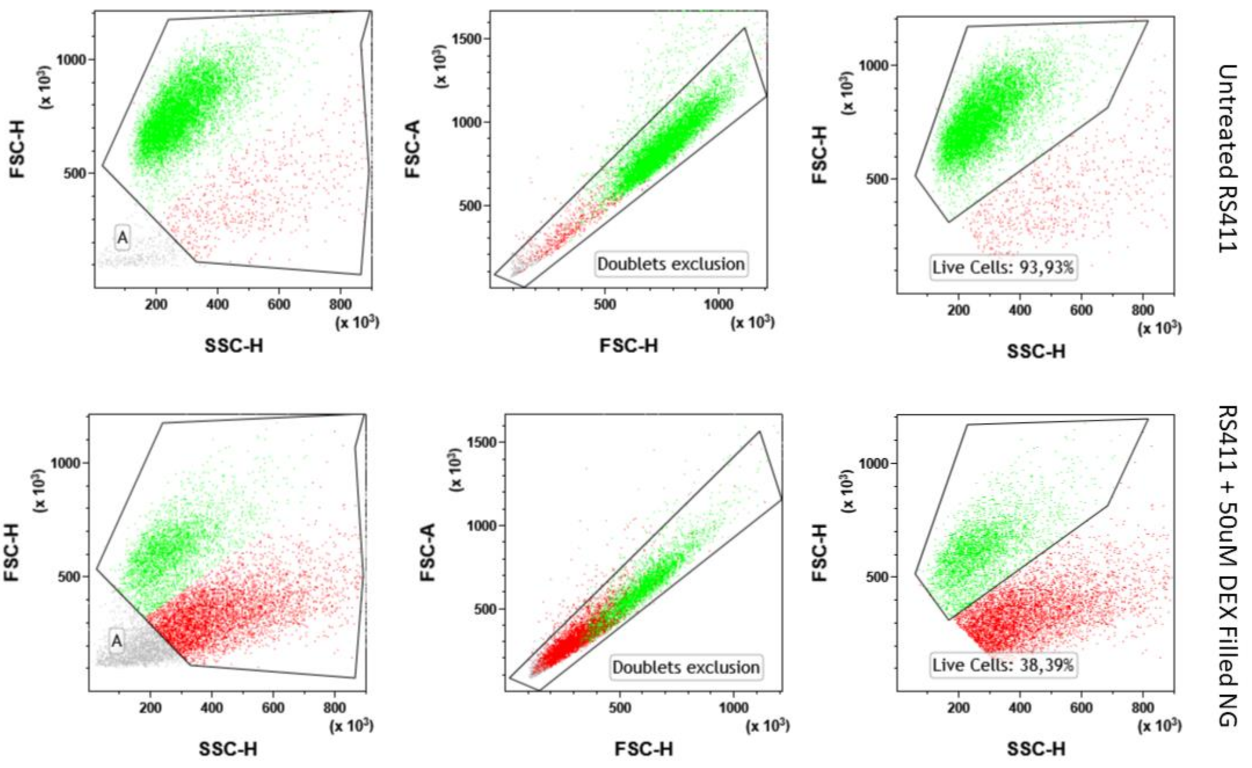

Figure S5. Flow Cytometry gating strategy to discriminate live cells. The FSC-H *vs* SSC-H plot to exclude debris; the FSC-A *vs* FSC-H plot to exclude doublets. After doublets exclusion, the FSC-H *vs* SSC-H plot was used to select Live cells.

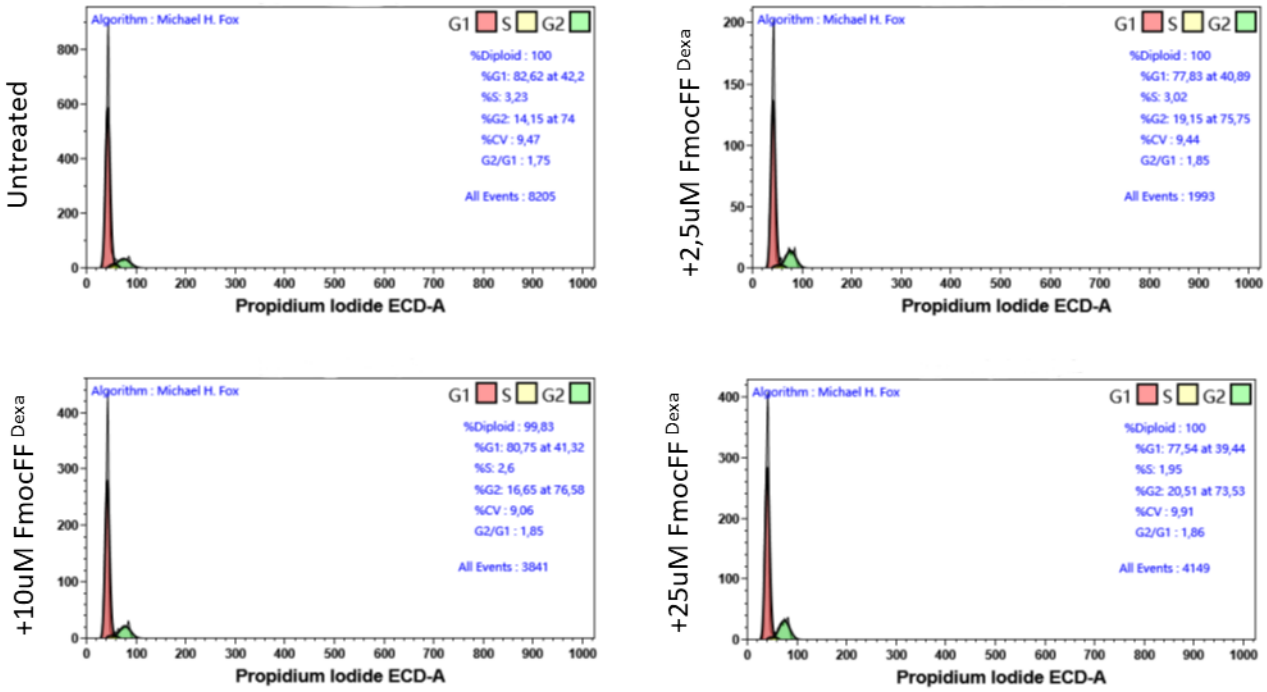

Figure S6. Flow cytometry analysis of the cell cycle distribution in untreated RS4;11 and RS4;11 treated with DEX filled NG at different concentrations (2,5, 10 and 25µM) after 24 hours of incubation. Cell cycle phases were calculated using the Michael H. Fox algorithm.

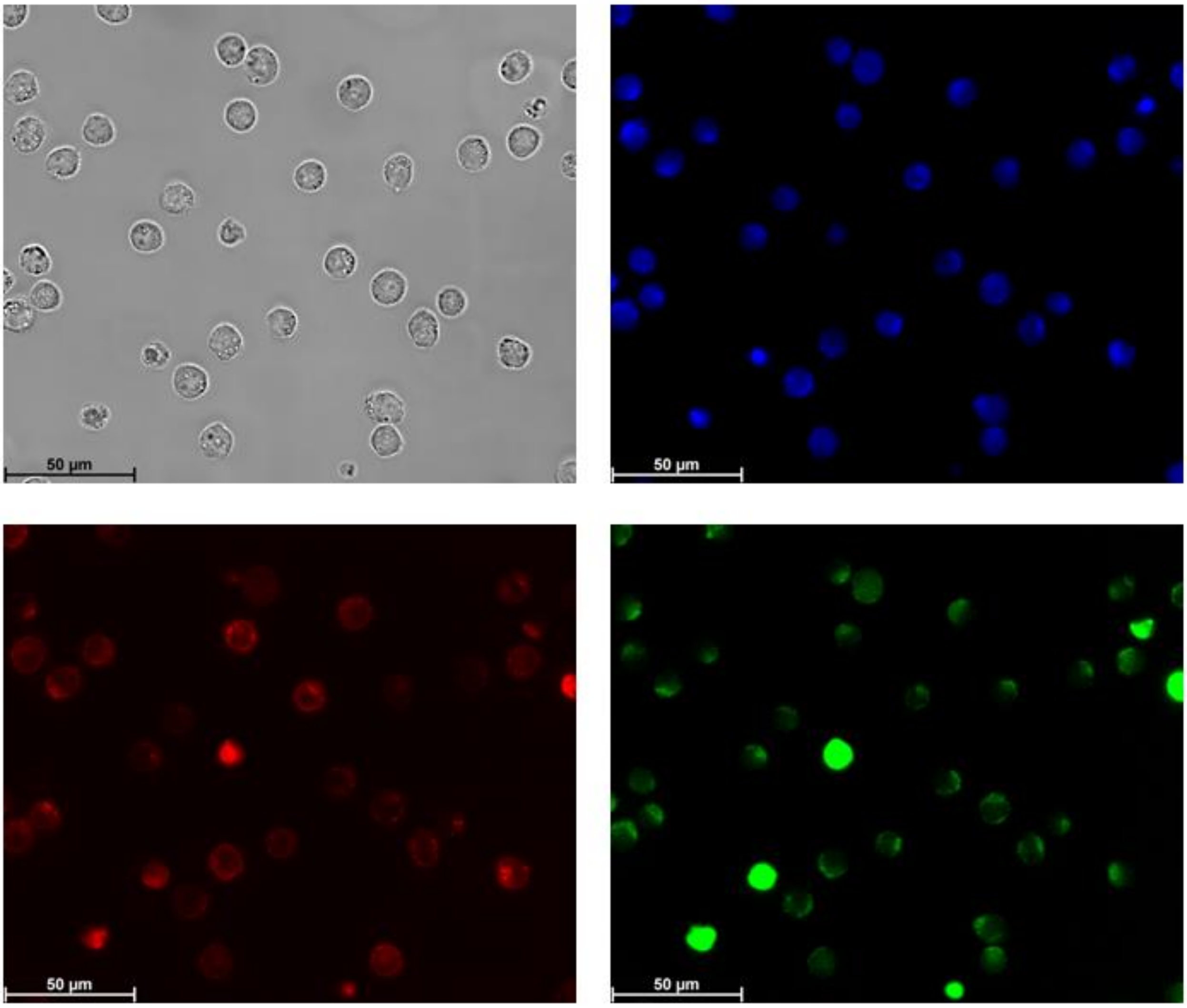

Figure S7. Fluorescence microscopy single channels of image reported in Figure 7. Upper left panel = Brightfield channel. Upper right panel = Hoechst channel. Lower left panel = HLA-DR-PC5 channel. Lower right panel = FITCS channel. Magnification 63x. Scale Bar 50µm.

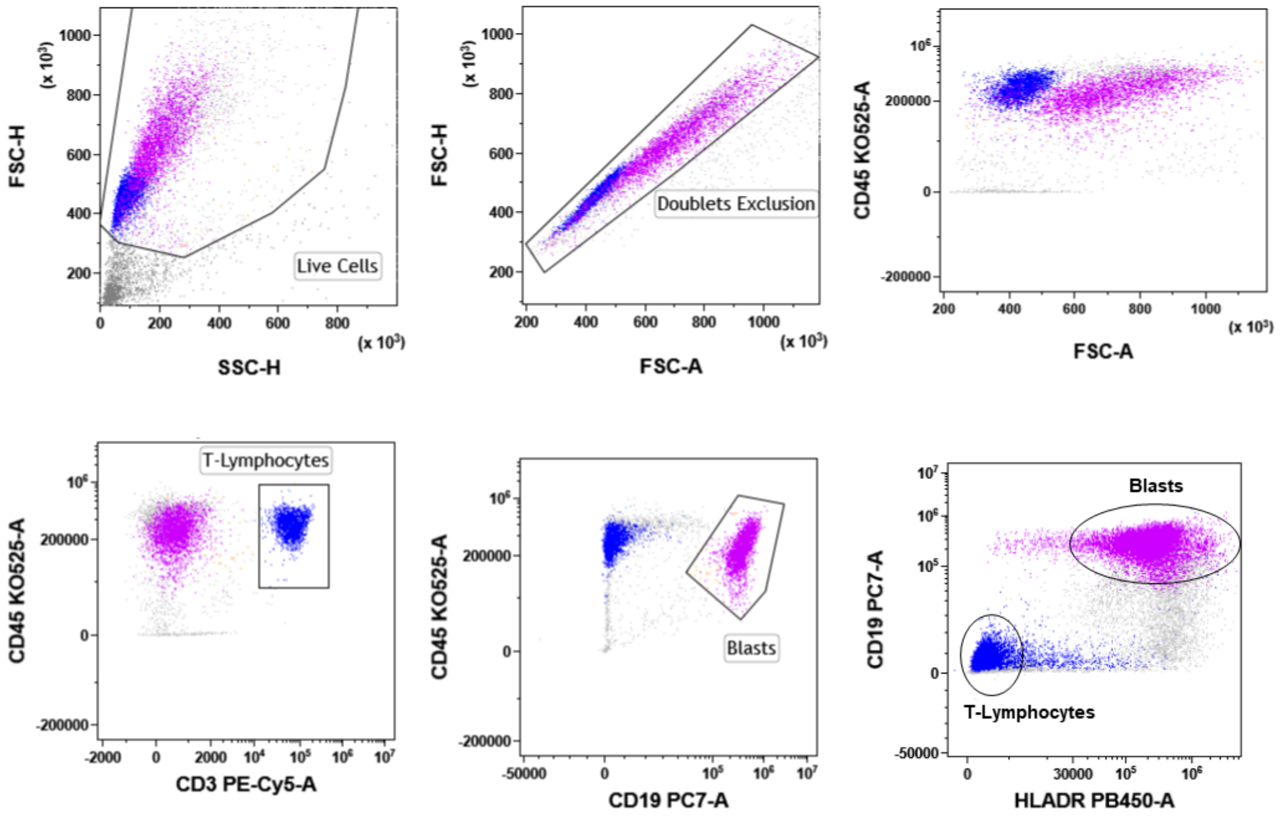

Figure S8. Flow cytometry gating strategy to discriminate between T-Lymphocytes (CD45pos/CD3pos) and RS4;11 cells (CD45pos/CD19pos/HLADRpos) cells. The FSC-A *vs* FSC-H plot to exclude doublets; FSC-H *vs* SSC-H to select live cells and exclude debris. CD45-KO-A *vs* CD3PC5-A to select T-lymphocytes; CD45-KO-A vs CD19-PC7-A to select RS4;11. CD19-PC7-A *vs* HLADR-PB-A to confirm the two subsets.
